# HIV-1 Tat expression drives progressive synaptic decline: evidence from longitudinal [^11^C]-UCB-J PET imaging

**DOI:** 10.1515/nipt-2025-0020

**Published:** 2026-02-17

**Authors:** Tara Chand Yadav, Isabella C. Orsucci, Spencer V. Thompson, Zhanhong Wu, Zibo Li, Hong Yuan, Barkha J. Yadav-Samudrala, Sylvia Fitting

**Affiliations:** Department of Psychology & Neuroscience, 2331 University of North Carolina at Chapel Hill, Chapel Hill, NC, 27599, USA; Biomedical Research Imaging Center, School of Medicine, University of North Carolina at Chapel Hill, Chapel Hill, NC, 27599, USA; Department of Radiology, School of Medicine, University of North Carolina at Chapel Hill, Chapel Hill, NC, 27599, USA

**Keywords:** HIV-1 Tat, synaptic density decline, SV2A, longitudinal PET imaging, [^11^C]-UCB-J, PSD-95

## Abstract

**Objectives:**

In the era of combined antiretroviral therapy (cART), human immunodeficiency virus type 1 (HIV-1) is considered a chronic condition that continues to affect the brain. As synaptodendritic injury occurs early, often years before the onset of clinical symptoms, noninvasive neuroimaging methods such as positron emission tomography (PET) hold promise for early detection.

**Methods:**

A doxycycline (DOX)-inducible HIV-1 Tat_1-86_ transgenic mouse model was used to examine the longitudinal impact of Tat expression on synaptic density *in vivo.* PET imaging with [^11^C]-UCB-J, a radiotracer targeting synaptic vesicle glycoprotein 2A (SV2A), was performed at 0-, 2-, and 12-weeks after Tat induction via DOX to assess progressive changes in synaptic density in the cerebrum. Complementary Western blot analyses were conducted at 12 weeks of Tat induction to measure SV2A and PSD-95 protein levels across the cortex, prefrontal cortex, striatum, and cerebellum.

**Results:**

Longitudinal [^11^C]-UCB-J PET imaging revealed a progressive decline of SV2A in the cerebrum of DOX treated Tat(+) mice compared to Tat(−) controls. Western blot analyses demonstrated a significant genotype x sex interaction in the cortex in which Tat expression reduced SV2A protein levels in females, without affecting males. Further, lower SV2A protein levels were noted overall in males relative to females in the cortex, prefrontal cortex, and cerebellum. No significant effects were detected for PSD-95 protein expression.

**Conclusions:**

These findings provide *in vivo* evidence that HIV-1 Tat expression contributes to progressive synaptic density decline and highlight sex-dependent differences in SV2A regulation, supporting SV2A as a biomarker for synaptic alterations in neuroHIV.

## Introduction

Human immunodeficiency virus type 1 (HIV-1) infects the brain and, despite combined antiretroviral therapy (cART), many people with HIV-1 (PWH) suffer from HIV-1-associated neurocognitive disorders (HAND) [1], [2], [3], [4]. A significant correlate of HAND progression is the appearance of synaptodendritic damage, including dendritic simplification, altered neurotransmission, and loss of viable synapses and synaptic connections [5], 6]. As alterations of synaptodendritic function in HAND and other neurodegenerative diseases occur early, years before the onset of clinical symptoms [5], [6], [7], it is important to develop and identify sensitive tools for early detection of such molecular changes.

Positron emission tomography (PET) imaging of synaptic vesicle glycoprotein 2A (SV2A), a protein located in the presynaptic vesicle membrane, that is ubiquitously expressed in virtually all synapses [8], 9], has emerged as a robust, noninvasive method to quantify synaptic density *in vivo* [10], [11], [12], [13]. Several PET tracers such as SV2A ligands have been developed since 2013 to quantify synaptic density in the living brain [14], [15], [16], [17], [18], [19]. Among them, [^11^C]-UCB-J has emerged as the most promising probe with high affinity and sufficient penetration to the blood-brain-barrier (BBB) [18], 20]. Initially validated in rhesus macaques, [^11^C]-UCB-J demonstrated robust uptake across grey matter regions, rapid kinetics, and high specificity for SV2A [18]. Subsequent studies confirmed its high specificity as an SV2A ligand in humans [21], minipigs [22], and rodents [23]. Since its development, [^11^C]-UCB-J PET imaging has been widely applied in both clinical [13], [24], [25], [26], [27], [28], [29], [30] and preclinical [31], [32], [33] studies, establishing SV2A as a sensitive biomarker for early synaptic alterations associated with a range of neuropathological conditions, including Alzheimer’s disease, Parkinson’s disease, epilepsy, spinal cord injury, and ischemic stroke. Additionally, several longitudinal [^11^C]-UCB-J PET studies have demonstrated progressive *in vivo* decline in synaptic density over the course of disease progression [34], 35], as well as treatment-induced normalization of initial synaptic deficits [33]. Notably, a recent cross-sectional pilot study reported reduced SV2A [^11^C]-UCB-J uptake in older PWH on suppressive cART compared to HIV-uninfected individuals [36], suggesting potential synaptic vulnerability in neuroHIV. Nevertheless, more research is necessary to determine whether these changes reflect cumulative neurotoxic effects of HIV, comorbidities, or ongoing viral protein exposure.

To address this gap, the present longitudinal study employed [^11^C]-UCB-J PET imaging to assess progressive changes in synaptic density in the HIV-1 Tat transgenic mouse model. The HIV-1 transactivator of transcription (Tat), a virally encoded regulatory protein, is known to be neurotoxic both *in vivo* [37], [38], [39], [40], [41], [42] and *in vitro* [43], [44], [45], [46], [47], [48], and continues to be detected in the brains of PWH despite suppressive cART [49], 50]. Importantly, Tat-induced central nervous system (CNS) abnormalities in this model recapitulate key neuropathological feature observed in PWH on cART, including dendritic simplification [37], 40], 51], altered neurotransmission [52], [53], [54], and reductions in synaptic vesicle and structural proteins [i.e. βIII Tubulin, synaptophysin, and postsynaptic density 95 (PSD-95)] [42], 51], 55], 56]. As SV2A and PSD-95 respectively represent presynaptic and postsynaptic markers of synaptic integrity [57], the current study tested the *a priori* hypothesis that Tat expression induces a progressive decline of SV2A density detectable by [^11^C]-UCB-J PET. Complementary *ex vivo* analysis further assessed Tat-induced alterations in SV2A and PSD-95 expression across cortical and subcortical brain regions. Together, these findings aim to elucidate Tat-mediated synaptic pathology and identify targets for mitigating HAND-associated neurodegeneration.

## Materials and methods

### Animals

HIV-1_IIIB_ Tat_1-86_ transgenic female and male mice on a C57BL/6J hybrid background [37], 39] were used in this study. In this inducible model, Tat expression is restricted to astrocytes in the brain via a glial fibrillary acidic protein (GFAP) promoter driving a reverse tetracycline transactivator (rtTA). Mice carrying the tetracycline response element (TRE)-tat transgene were identified by Transnetyx, Inc. (Cordova, TN) and designated as Tat(+) animals, while littermates lacking the transgene served as Tat(−) controls. To induce Tat expression, Tat(+) mice received doxycycline (DOX) chow (6 mg/g; TD.09282, Inotiv, Indianapolis, IN) *ad libitum*; Tat(−) controls were given the same DOX-containing diet to control for off-target effects. Prior to Tat induction, all mice were maintained on standard chow (IsoPro RMH 3000, Purina LabDiet, St. Louis, MO). Animals were group-housed (3–5 per cage) under a reversed 12-h light/dark cycle (lights off at 6:00 a.m.) with free access to food and water.

**Figure 1:**
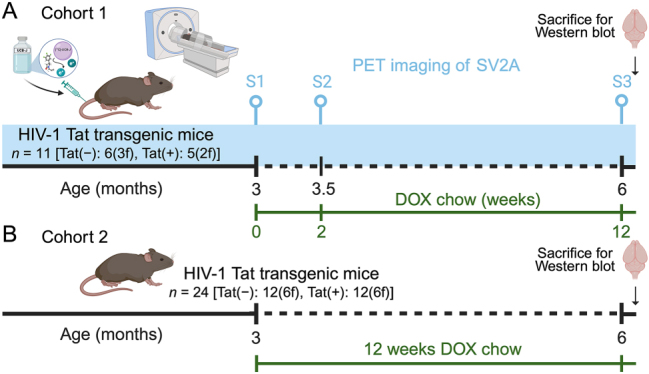
**Experimental design.** (A) HIV-1 Tat transgenic mice in cohort 1 (n=11) underwent longitudinal PET/CT imaging using the SV2A radiotracer [^11^C]-UCB-J at baseline (no DOX), 2-weeks DOX, and 3-months DOX chow to induce Tat expression. Following the final PET scan, brains were collected for subsequent analyses. (B) An age-matched cohort (cohort 2, n=24) underwent 12-weeks DOX exposure and was used for regional SV2A and PSD-95 quantification by Western blot. Mice from cohort 1 were included in the Western blot dataset. PET, positron emission tomography; SV2A, synaptic vesicle glycoprotein 2A; DOX, doxycycline; S1, session 1; S2, session 2; S3 session 3.

This study included two cohorts of mice (Figure 1). The first cohort (Figure 1A) included Tat(+) and Tat(−) mice (n=6, 3 females per group) for longitudinal PET imaging; one Tat(+) female was excluded due to a head movement artifact, leaving 5 mice (2 females) in the Tat(+) group. The second cohort (n=12(6f) per group, Figure 1B) was used for western blot analysis to validate the PET imaging findings after 3-months DOX exposure. Western blot assays were also performed on the first cohort for that time point, with data combined for presentation with the second cohort.

All procedures complied with the NIH Guide for the Care and Use of Laboratory Animals (NIH Publication No. 85–23) and were approved by the University of North Carolina at Chapel Hill Institutional Animal Care and Use Committee (IACUC, #20-108.0).

### Positron emission tomography (PET) imaging

#### Radioligand synthesis of [^11^C]-UCB-J

The synthesis of [^11^C]-UCB-J was performed as previously described [18]. The [^11^C]-UCB-J probe was produced at the Biomedical Research Imaging Center (BRIC) Cyclotron and Radiochemistry Core facility according to the well-established radiosynthesis method [18]. In brief, the imaging probe was synthesized by radiomethylation of the phenolic precursor with [^11^C]methyl iodide. The product was purified by high-performance liquid chromatography (HPLC) and formulated in sterile saline with <10 % ethanol. Radiochemical purity exceeded 99 % and the molar activity was 463.61 ± 108.78 GBq/μmol.

#### Dynamic [^11^C]-UCB-J PET imaging

All animal PET and computed tomography (CT) imaging was conducted in the BRIC Small Animal Imaging (SAI) facility. PET/CT image acquisition was performed using a small animal PET/CT scanner (SuperArgus-4R, Sedecal, Inc. Spain) with a spatial resolution of 1.0 mm in the center field of view (FOV). Animals were anesthetized using isoflurane inhalation (1.5–2.0 % mixed with oxygen). Four mice (including both genotypes and both sexes) were scanned per session using a house-built 4-bed mouse holder. Each animal was secured to the bed with an individual respiration sensor to monitor the respiration rate during imaging. Prior to positioning, a tail vein catheter was placed on the tail of each animal for radiotracer injection. A dose of [^11^C]-UCB-J (average 12.9 ± 2.2 MBq in 100 μL saline) was administered through mouse tail vein. Dynamic PET imaging was immediately started following the start of the injection and continued for 65 min. CT imaging was performed right before PET for attenuation correction and anatomical reference. Raw PET images were first binned (6 × 10 s, 4 × 30 s, 2 × 60 s, 3 × 5 min, 4 × 10 min, 1 × 5 min) into 20 frames and were reconstructed using the 3D- ordered subject expectation maximization (OSEM) algorithms with scatter, attenuation, and decay correction, to obtain dynamic PET images with voxel size of 0.37 × 0.37 × 0.78 mm. No partial volume correction was applied during or post PET reconstruction, thus quantification accuracy in small regions is largely limited by low resolution of PET imaging. At the end of the PET/CT scan, animals were recovered from anesthesia and brough back to the housing room for full radiation decay. Each mouse was imaged at three time points: baseline prior Tat induction (no DOX; session 1, S1), 2-weeks post Tat induction (session 2, S2), and 3-months post Tat induction (session 3, S3), as shown in Figure 1A.

#### PET image analysis

PET and CT images were processed and analyzed using PMOD software (Version 4.0, Bruker Biospin, Billerica, Massachusetts, USA). PET images were first registered to the CT images using the established matrix transformation. The volume of interest (VOI) of cerebrum was segmented in the CT images and defined as the brain region extending 6 mm in the rostral-caudal direction from the end of the olfactory bulb to the beginning of the cerebellum. The VOI was then superimposed on the PET images to extract the time-activity curves (TAC) of the defined brain region. The cerebrum was selected as the VOI due to its known vulnerability to HIV- and Tat-associated synaptic dysfunction, its relevance to cognitive and behavioral outcomes, and its suitability for reliable PET-based quantification of synaptic density. Muscle in the upper arm was chosen as the reference tissue, because there was high uptake in the cerebellum tissue which is otherwise used as the reference tissue. Standardized uptake value (SUV) of each tissue was obtained by normalizing the activity uptake in tissue to the injected dose and body weight. The SUV ratio (SUVR) between cerebrum and the muscle tissue at 25 min, when brain uptake reached peaked, was calculated and compared across baseline, 2-weeks, and 3-months post Tat induction time points. Since each animal had a baseline PET/CT imaging to serve as self-control, the percentage change of SUVR (%Change) relative to its baseline was further calculated for each animal.

### Western blot

The western blot assay was carried out on four brain regions of female and male HIV-1 Tat transgenic mice (3-months DOX exposure), including the cortex, prefrontal cortex, striatum and cerebellum as previously described [58], [59], [60], [61]. Briefly, tissue samples were homogenized using sufficient ice-cold Pierce™ RIPA lysis buffer (Thermo Scientific, Cat# 89901, Waltham, MA, USA) containing Halt™ protease and phosphatase inhibitor cocktail. The Pierce™ BCA protein assay kit (Thermo Scientific, Cat# 1859078) was used to determine protein concentration in the tissue lysates. Protein lysates were denatured for 10 min at 85 °C. Equivalent quantities of protein (20 μg/lane) were resolved via SDS-PAGE at 120 V for 1.5 h and transferred to nitrocellulose membranes (Biorad, Cat# 1620115) at 1–4 °C and 100 V for a duration of 1 h. Membranes were blocked with Intercept^®^ blocking buffer (LI-COR Biosciences, Cat# 927–70001, Lincoln, NE, USA) for 1 h at room temperature, and then incubated with primary antibodies, anti-SV2A (rabbit polyclonal; Abcam, Cat# ab32942, 1:10,000 dilution, Cambridge, UK), anti-PSD-95 (rabbit polyclonal; Invitrogen, Cat# MA10145, 1:10,000 dilution), and anti-GAPDH antibody as a loading control (mouse monoclonal; Abcam, Cat# ab125247, 1:10,000 dilution) at 4 °C overnight. Next day, membranes were incubated with secondary antibodies, IRDye^®^ 680RD Donkey anti-Mouse IgG (LI-COR Biosciences, Cat# 926–68072, 1:15,000 dilution) and IRDye^®^ 800CW Donkey anti-Rabbit IgG (LI-COR Biosciences, Cat# 926–32213, 1:15,000 dilution) at room temperature for 1 h in Intercept^®^ blocking buffer. Membranes were imaged with an Odyssey^®^ CLx infrared imaging system (LI-COR Biosciences) and analyzed using Empiria studio^®^ software version 2.3.0 (LI-COR Biosciences). Data reported are normalized to the housekeeping gene GAPDH, with fold change calculated using a reference control sample on all blots to ensure consistency across analyses. A positive control was used for all Western blot gels, which were run in lane 1.

### Statistical analysis

For Western blot data, statistical significance was assessed by two-way analysis of variances (ANOVAs) with genotype [2 levels: Tat(−) mice, Tat(+) mice] and sex (2 levels: females, males) as between-subjects factors followed by Fisher’s LSD post hoc tests when appropriate (SPSS Statistics 25; IBM, Chicago, IL, USA). Additionally, a repeated one-way ANOVA was performed for brain regions (4 levels: cortex, prefrontal cortex, striatum, cerebellum) as a within-subjects factor followed by Fisher's LSD post hoc tests when appropriate, to assess differences in synaptic protein expression across brain regions. For the SUVR data from PET imaging, three-way mixed ANOVAs were conducted with DOX treatment (3 levels: no DOX, 2-weeks DOX, 3-months DOX) as a within-subjects factor and genotype and sex as between-subjects factor. ANOVA results are reported as within-subjects contrasts to test the *a priori* hypothesis that [^11^C]-UCB-J uptake declines over time with DOX exposure in Tat(+) mice due to Tat induction compared to Tat(−) controls. Significant interactions were addressed by repeated ANOVAs for each genotype followed by Fisher’s LSD post hoc tests for multiple comparison. The relationship between SUVR PET data and Western blot data of SV2A protein expression was assessed via Pearson correlation. Differences of p-values ≤0.05 were considered statistically significant. All data are presented as mean ± the standard error of the mean (SEM).

## Results

### [^11^C]-UCB-J PET imaging of SV2A

#### SUVR of [^11^C]-UCB-J detects progressive decline of SV2A in the cerebrum

**Figure 2:**
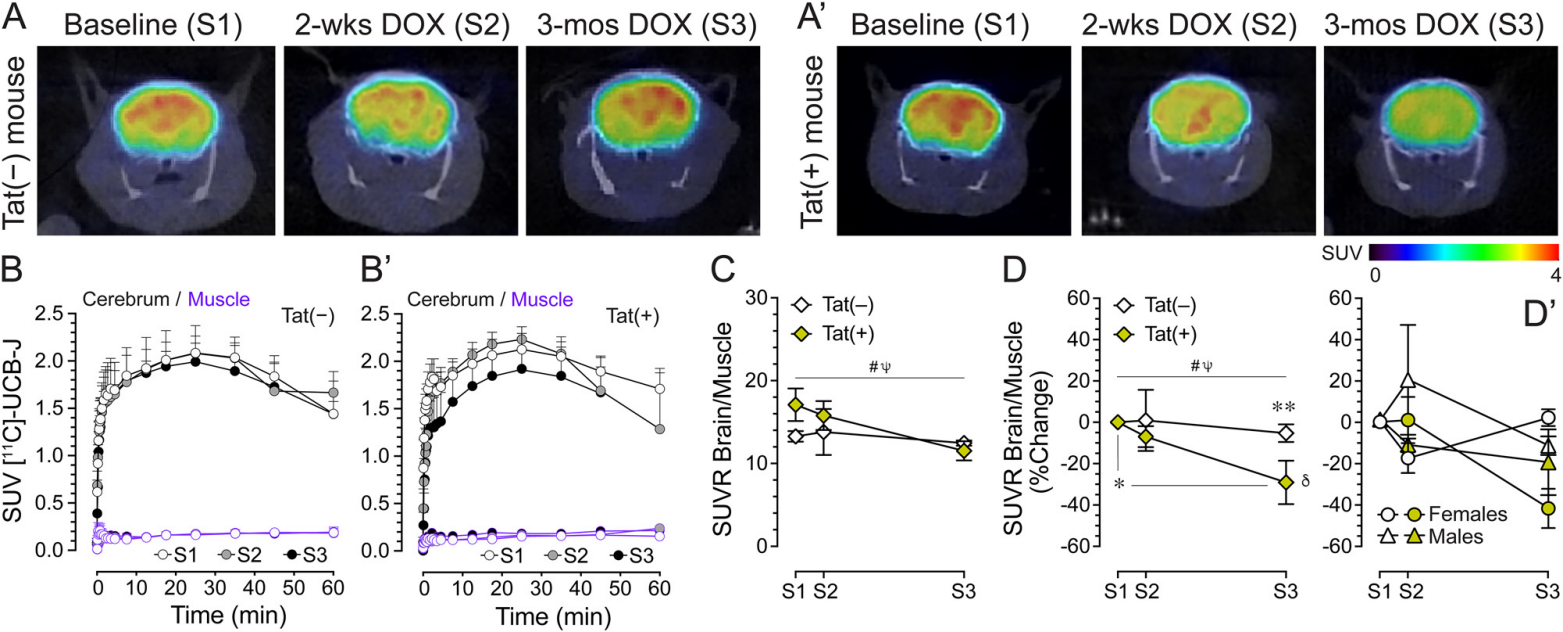
**[**^**11**^**C]-UCB-J PET reveals progressive SV2A loss in the cerebrum.** (A, A') Representative coronal PET/CT images (20–30 min post injection) from Tat(−) and Tat(+) mice at baseline (no DOX), 2-weeks DOX, and 3-months DOX. (B, B′) Cerebral time activity curves across the three sessions for Tat(−) and Tat(+) mice. (C) Mean cerebrum/muscle SUVR showed a significant decline over time, modulated by genotype, with no between-group differences at individual time points. (D) Percent change from baseline SUVR revealed a progressive reduction in Tat(+) but not Tat(−) mice; (D′) no effects for each sex were observed. Data are expressed as mean ± SEM. Circles represent individual female data points; triangles represent individual male data points. Statistical significance was assessed by ANOVAs followed by Fisher’s LSD post hoc tests. ^*#*^p<0.05, linear main effect of DOX treatment; ^*Ψ*^p<0.05, linear DOX treatment x genotype interaction; ^*δ*^p=0.05, main effect of DOX treatment in Tat(+) mice; ^*^p=0.05, S1 versus S3 for Tat(+) mice; ^**^p=0.01, Tat(+) mice versus Tat(−) mice at S3. PET, positron emission tomography; S1, session 1; S2, session 2; S3, session 3; DOX, doxycycline; wks, weeks; mos, months; SUV, standardized uptake values; TAC, time-activity curves; SUVR, ratio of standard uptake values. n=5–6 per genotype.

Dynamic [^11^C]-UCB-J PET imaging was conducted in Tat transgenic mice *in vivo* [11 mice, n=5–6(2–3f) per genotype] to assess SV2A density at no DOX (baseline, S1), 2-weeks DOX (S2), and 3-months DOX (S3) treatment within the same mice (Figure 2). Representative [^11^C]-UCB-J PET/CT images at 25 min post injection are shown in Figure 2A, A' for a Tat(−) mouse and Tat(+) mouse across the three sessions. The tissue activity curves (TAC) during a 60-min imaging period at baseline (S1), 2-weeks DOX (S2), and 3-months DOX (S3) are shown for Tat(−) mice (Figure 2B) and Tat(+) mice (Figure 2B′). A rapid and high uptake of the [^11^C]-UCB-J tracer was observed immediately after the i.v injection of the radiotracer, achieving peak levels at 25-min post injection followed by a steady reduction to 60 min. Based on the TAC uptake pattern, the SUV at the 25-min mark was used to represent tracer uptake in the cerebrum. SUV brain/muscle ratio (SUVR) at 25 min was calculated using muscle tissue as its internal reference.

For SUVR of the [^11^C]-UCB-J SV2A tracer across the three sessions, a three-way ANOVA demonstrated a progressive downregulation of SV2A expression over the three sessions (linear DOX treatment effect, p=0.02; Figure 2C), which was altered by genotype (linear DOX treatment x genotype interaction, p=0.05). SV2A expression appeared to decline to a greater extent in Tat(+) mice compared to Tat(−) mice, however one-way repeated ANOVAs for each genotype revealed no significant time effect, likely due to high intersubject variation. As, no significant genotype differences were found at baseline (S1) and to reduce the intersubject variation, SUVR (%Change) relative to its baseline was compared at 2-weeks DOX and 3-months DOX treatment for both genotypes. A three-way ANOVA for SUVR (%Change) across the three sessions revealed a progressive decline of SV2A expression over the three sessions (linear DOX treatment effect, p<0.01; 3 % S1 vs. S2; 16 % S1 vs. S3; Figure 2D). Importantly, the significant linear DOX treatment x genotype interaction (p=0.04), indicated that SV2A expression declined more rapidly in Tat(+) mice [repeated one-way ANOVA, p=0.05; 7 % S1 versus S2; 29 % S1 versus S3, LSD: p=0.05] compared to their Tat(−) controls (n.s.; 1 % S1 vs. S2; 5 % S1 vs. S3). Comparing both genotypes at 3-months DOX, Tat(−) controls showed less SV2A reduction (5.27 % ± 4.25) compared to Tat(+) mice (29.10 % ± 10.57, LSD: p=0.05). No significant effects or interactions were noted for sex (Figure 2D’).

### Quantification of SV2A and PSD-95 via Western blot analysis

#### SV2A protein levels are reduced by Tat expression in the cortex

**Figure 3:**
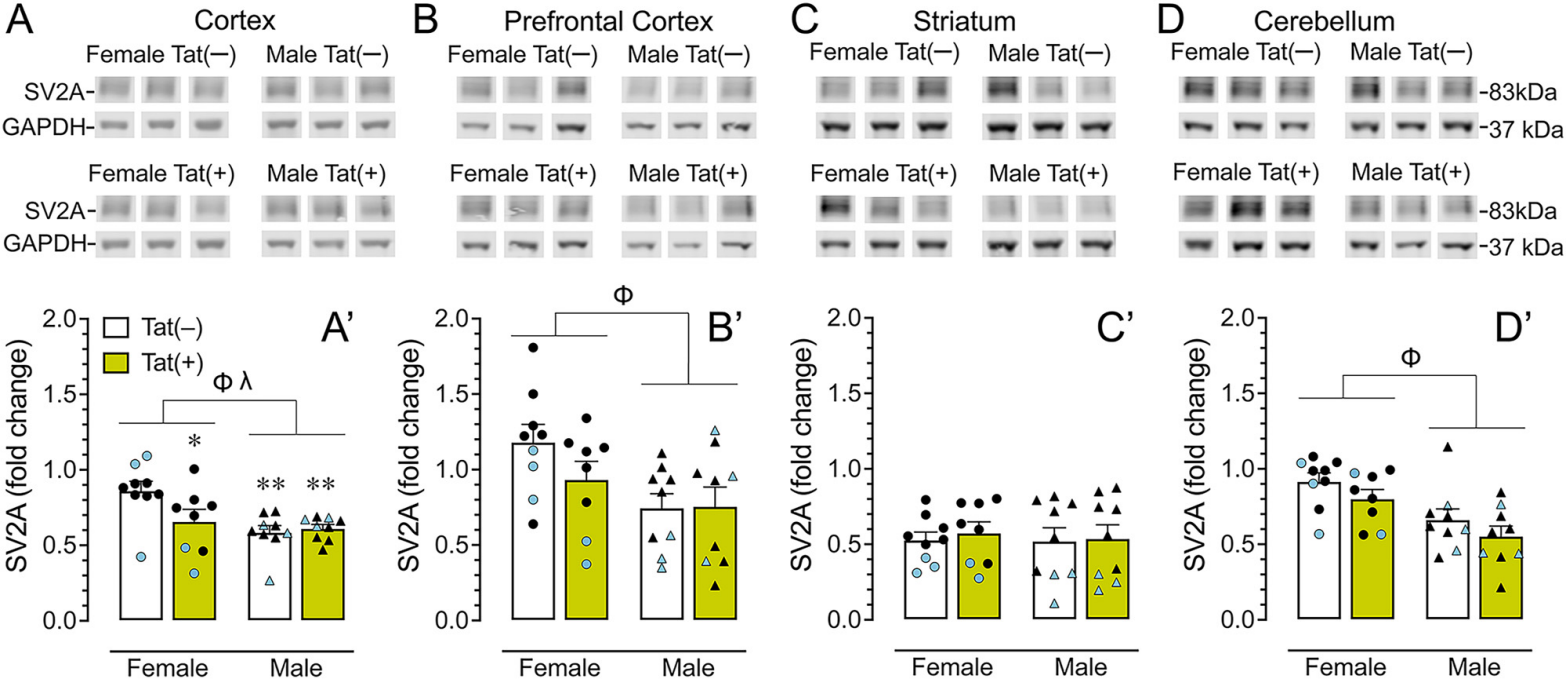
**Western blot analysis reveals reduced cortical SV2A expression in female mice with Tat expression.** SV2A protein levels were quantified in female and male mice following 3-months DOX exposure. Data were normalized to GAPDH and expressed as fold change relative to a control sample included on all blots. Representative immunoblots for SV2A and GAPDH are shown for each group across four brain regions. (A, A′) In the cortex, females exhibited higher SV2A expression compared to males, with Tat expression selectively reducing cortical SV2A in females. (B, B′) In the prefrontal cortex, female mice showed higher SV2A protein levels than males. (C, C′) In the striatum, SV2A expression did not differ across groups. (D, D′) In the cerebellum, females demonstrated higher SV2A expression than males. Data are expressed as mean ± SEM. Closed circles (blue colored represent PET subjects) and closed triangles (blue colored represent PET subjects) represent individual data points of female and male mice, respectively. Two-way ANOVAs were performed followed by Fisher’s LSD post hoc tests when appropriate. ^*Φ*^p<0.05, sex main effect; ^λ^p<0.05, sex x genotype interaction. Fisher’s LSD: *p<0.05 versus Tat(−) female, **p<0.01 versus Tat(−) female. n=8–9 per genotype.

Four brain regions were assessed to evaluate the effects of Tat expression on SV2A protein expression (Figure 3). Representative bands from each group for the cortex, prefrontal cortex, striatum, and cerebellum are displayed in Figure 3A–D. Band intensities were normalized to the housekeeping protein GAPDH, with fold change calculated using a reference control sample on all blots to ensure consistency across analyses. A one-way repeated ANOVA for SV2A expression demonstrated significant differences between brain regions (p<0.001), with all brain regions significantly differing from each other (LSD: p’s<0.01), except for cortex compared to cerebellum. The order of SV2A expression from highest to lowest was as follows, prefrontal cortex>cortex=cerebellum>striatum.

For the cortex (Figure 3A′), a two-way ANOVA revealed a significant sex effect (p<0.01), with males exhibiting lower SV2A protein levels compared to females. More importantly, genotype significantly influenced the sex effect (sex x genotype interaction, p<0.05), with Tat expression for females significantly reducing SV2A protein levels compared to Tat(−) females (p=0.02), whereas this was not seen for male mice. Similarly to the cortex, lower SV2A protein levels were noted for males compared to females in the prefrontal cortex (Figure 3B′, p=0.01) and cerebellum (Figure 3D′, p<0.001). No significant effects were observed for the striatum (Figure 3C′). Correlation analysis in cohort 1 mice that underwent both PET imaging and Western blot analysis (n=11) revealed a significant positive association between cerebrum SUVR and SV2A protein expression in the prefrontal cortex (*r*=0.619, *R*^*2*^=0.393, p=0.04), whereas no significant correlations were observed with SV2A protein levels in the cortex or striatum. Notably, this association was driven by Tat(+) mice (*r*=0.950, *R*^*2*^=0.902, p=0.01, n=5) and was not present in Tat(−) mice (*r*=−0.118, *R*^*2*^=0.014, p=0.82, n=6).

### PSD-95 protein levels are not affected by Tat expression

**Figure 4:**
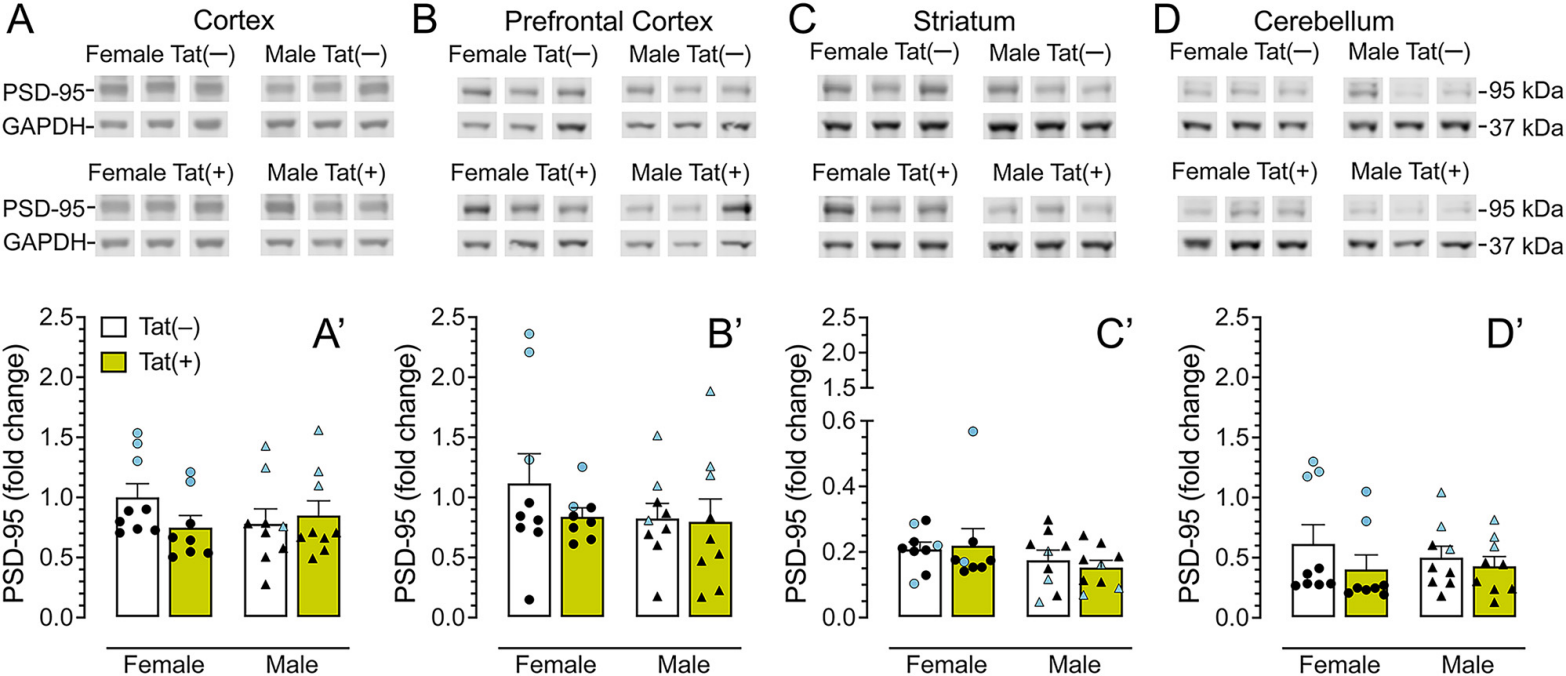
**PSD95 protein expression assessed by Western blot analysis is not affected by Tat expression or sex.** PSD-95 levels were quantified in female and male mice following 3-months DOX exposure. Data were normalized to GAPDH and expressed as fold change relative to a control sample included on all blots. Representative immunoblots for PSD-95 and GAPDH are shown for the cortex (A, A′), prefrontal cortex (B, B′), striatum (C, C′), and cerebellum (D, D′). No significant differences were observed in any region. Data are expressed as mean ± SEM. Closed circles (blue colored represent PET subjects) and closed triangles (blue colored represent PET subjects) represent individual data points of female and male mice, respectively. Two-way ANOVAs were performed to assess statistical significance. n=8–9 per genotype.

PSD-95 protein levels are shown in Figure 4. Representative bands from each group for the cortex, prefrontal cortex, striatum, and cerebellum are displayed in Figure 4A–D. A one-way repeated ANOVA for SV2A expression demonstrated significant differences between brain regions (p<0.001), with all brain regions significantly differing from each other (LSD: p’s<0.001) compared to prefrontal cortex, except for cortex. The order of PSD-95 expression from highest to lowest was as follows, cortex=prefrontal cortex>cerebellum>striatum.

No significant sex or genotype effects were observed in any brain region (Figure 4A′–D′).

## Discussion

Synaptic injury and degeneration is a critical pathological hallmark of cognitive impairment in PWH, as evidenced by reduced levels of synaptic proteins such as synaptophysin, synaptotagmin, and PSD-95 in post-mortem brain tissues of PWH [47], 51]. A recent SV2A [^11^C]-UCB-J PET imaging study further provided *in vivo* evidence of significantly lower synaptic density in PWH on cART in the frontostriatalthalamic circuit that was associated with poorer performance in motor and executive functions [36]. The findings of the present longitudinal study confirm the decrease of [^11^C]-UCB-J SV2A uptake in a well-established neuroHIV mouse model. Specifically, a progressive loss of synaptic density in the cerebrum of HIV-1 Tat transgenic mice was observed upon Tat induction over a 3-months period. Additionally, complementary Western blot analysis found Tat-induced downregulation of SV2A protein expression in the cortex of female mice, with Tat expression not affecting PSD-95 protein levels in any brain region. Together, PET imaging with [^11^C]-UCB-J and complementary protein analyses support the utility of SV2A as a biomarker for synaptic alterations in neuroHIV and suggest potential sex-specific vulnerabilities relevant to HAND pathogenesis.

A unique aspect of using the HIV-1 Tat transgenic mouse model is its ability to isolate Tat-specific neurotoxic effects independent of viral replication or peripheral comorbidities. This distinction is particularly important given that, despite suppressive cART, Tat continues to be released from infected cells and is detected in brains of PWH [49], 50]. As such, Tat remains a critical driver of synaptic injury and neurodegeneration, contributing to the persistent cognitive impairment observed in virally suppressed PWH [62], 63]. By selectively inducing Tat expression, this model provides a controlled system to examine Tat-mediated mechanisms of synaptic loss and to assess the translational potential of SV2A as an *in vivo* biomarker of synaptic density in neuroHIV.

Preclinical studies using the HIV-1 Tat transgenic mouse model have demonstrated synaptic alterations detectable as early as 1–2 weeks [51], 64] and persisting up to 2–3 months [42], 56], 65] following Tat induction, as assessed using *ex vivo* approaches such as immunohistochemistry and Western blot analyses. While many studies report downregulation of neuronal and synaptic markers such as βIII-tubulin, synaptophysin, and PSD-95 [42], 56], 66], others show unchanged or even increased expression of proteins like PSD-95, synaptophysin, synaptotagmin-2, and gephyrin [51], 56], 65], suggesting that Tat-induced synaptic pathology is temporally dynamic and characterized by an imbalance between pre- and postsynaptic elements rather than uniform synaptic loss. Notably, acute Tat exposure has been shown to inhibit vesicular monoamine transporter 2 (VMAT2)-mediated dopamine uptake in isolated vesicles and transgenic mice, indicating that Tat disrupts presynaptic vesicular dopamine handling [67], which may contribute to early synaptic dysfunction prior to detectable changes in structural synaptic proteins.

In the present longitudinal PET imaging study, we observed a progressive Tat-dependent decline in SV2A binding over a 3-month period. Specifically, after 2-weeks DOX exposure, a non-significant trend toward reduced SV2A uptake (∼7 %) was observed in Tat(+) mice compared to Tat(−) mice, whereas at 3-months DOX, this difference increased to a significant decline of 29 % in Tat(+) mice versus 5 % in Tat(−) mice. The lack of statistical significance at the early time point may reflect the low sample size and inter-subject variability, highlighting the need for further investigation to determine whether SV2A PET imaging can detect early synaptic alterations in neuroHIV. Importantly, the limited number of animals in the PET cohort also restricts power to detect subtle or sex-specific effects. Thus, the absence of significant changes at the 2-week time point and the lack of detectable sex differences should not be interpreted as evidence of biological absence, but rather as reflecting sensitivity limitations inherent to this study. Larger longitudinal PET studies will be required to definitively assess early synaptic changes and sex-dependent trajectories of SV2A loss in response to Tat expression.

Interestingly, the progressive *in vivo* decline in synaptic density in the current study occurred despite prior evidence suggesting Tat exposure induces a state of innate immune tolerance [68], with lack of microglial reactivity and reduced levels of specific cytokines (e.g., IL-1α, IL-12p40), which have been shown to be elevated by shorter duration (28-day) of Tat induction [69]. This dissociation suggests that neurons may be unable to fully recover from prior Tat-induced injury, and that Tat continues to exert lasting neurotoxic effects on synaptic integrity independent of ongoing inflammation. Such findings underscore the multifactorial nature of Tat-mediated neurodegeneration and highlight the need to consider both inflammatory and non-inflammatory mechanisms in efforts to preserve synaptic health in neuroHIV.

Our dynamic PET imaging data demonstrated rapid uptake of [^11^C]-UCB-J into brain tissue, suggesting efficient BBB penetration. Given the stable uptake of [^11^C]-UCB-J in mouse brain with a peak around 25 min post injection and a slow washout, SUVs at the 25-min time point were used to represent tracer uptake. To account for substantial inter-subject variability in SUV measures, muscle was selected as an internal reference tissue for SUV ratio (SUVR) calculations, minimizing effects from injection doses and plasma availability in tracer uptake. Although the cerebellum has been used as reference tissue in other studies [70], our imaging data showed rapid uptake followed by gradual decline in cerebellum, suggesting a dynamic process that makes it less suitable as a stable reference. As we could not identify a reliable low-binding region in the CNS, muscle tissue was selected as reference since it consistently showed low tracer uptake as indicated in Figure 2B, B′. Additionally, we want to highlight that SUVR percentage change was used to assess SV2A decline across sessions, as no significant baseline differences between genotype was observed, despite a trend for higher baseline values in Tat(+) mice. The measurement of absolute SUV and SUVR also showed large variations at baseline among all animals, indicating potential large individual differences in SV2A expression. Future studies with larger sample sizes are warranted to improve statistical power, particularly for assessing sex differences and evaluating potential therapeutic interventions. We further acknowledge that, given the dynamic uptake characteristics of the tracer, kinetic modeling would be the ideal method, as it measures the volume of distribution to assess tissue uptake across time points and genotypes. However, we encountered challenges in obtaining accurate arterial input function from the blood TACs, including acquisition errors resulting in missing early (first-minute) data and motion artifacts during imaging sessions. Consequently, only a limited number of animals had high-quality blood data across all three imaging sessions, preventing meaningful comparisons with sufficient statistical power. Future studies will be needed to characterize SV2A [^11^C]-UCB-J tissue uptake in this neuroHIV mouse model using dynamic modeling.

It is important to note that, due to the lack of kinetic modeling using arterial input functions, our SUVR measurements should be interpreted as relative changes rather than absolute quantification of SV2A expression. While SUVR provides a practical and robust approach for longitudinal comparisons within subjects, it is inherently sensitive to inter-subject variability in tracer delivery and reference tissue dynamics. This limitation constrains the quantitative rigor of the present study. Kinetic modeling in mouse models involving fast-clearing tracers is challenging due to measurement errors in reference tissue and image-derived input functions [71]. As previously demonstrated, the accurate estimation of V_T_ is sensitive to multiple technical factors, including reconstruction algorithms, image resolution, and attenuation and scatter corrections [71]. Future work will focus on optimizing dynamic acquisition and reconstruction protocols to improve image resolution, and to obtain reliable blood input functions in mouse models, enabling full kinetic modeling (e.g., calculation of volume of distribution, V_T_) to achieve more robust and quantitative assessment of SV2A density in this model. It is also worth noting that kinetic modeling analysis requires long scan time and analysis time. Some simplified quantification using SUVR at an optimal time window in a well-defined cohort has been evaluated in human subjects with promising results [72].

Given the progressive decline in SV2A [^11^C]-UCB-J uptake observed through SUVR analyses, we employed a complementary approach to further investigate these findings. To increase statistical power, we included an additional age-matched cohort of HIV-1 Tat transgenic mice treated with DOX for 3 months, alongside those used for PET imaging, to assess SV2A and PSD-95 protein expression via Western blot. This approach allowed us to examine both sex differences and regional specificity across multiple brain regions, including the cortex, prefrontal cortex, striatum, and cerebellum. Interestingly, Tat expression significantly reduced SV2A protein levels selectively in female mice and specifically within the cortex, with no detectable Tat effects in males across all regions examined. Moreover, females exhibited higher overall SV2A expression than males across all regions examined, except for the striatum. These findings are consistent with prior reports demonstrating sex-dependent effects of Tat on synaptic markers, in which both the direction and magnitude of Tat-induced changes vary as a function of brain region and duration of Tat expression [56], 66]. Although, several studies have reported Tat-associated alterations in synaptic protein expression in male mice [51], 55], 56], our data suggest that presynaptic SV2A within the cortex may be particularly vulnerable in females. This heightened susceptibility may be influenced by sex-specific hormonal regulation, as estrogens and other gonadal hormones are known to modulate Tat’s effects on synaptic integrity. Indeed, Tat has been shown to disrupt hypothalamic-pituitary-adrenal (HPA) and hypothalamic-pituitary-gonadal (HPG) axis function more profoundly in females [73], potentially contributing to altered synaptic balance. Consistent with this interpretation, Tat exposure has been associated with greater increases in inhibitory synaptic neurotransmission in females, including elevated spontaneous inhibitory postsynaptic current frequency in the prefrontal cortex [53], as well as pronounced prefrontal cortex-dependent behavioral alterations compared to males [38], 58], 60]. Notably, PSD-95 levels remained unchanged across all regions and sex in our study, consistent with previous studies in the hippocampus after 2-weeks DOX exposure [51] and in the anterior cingulate cortex after 2-months DOX exposure [55]. The observed reduction in SV2A, but not PSD-95, likely reflects the greater vulnerability to the presynaptic compartment to Tat-induced neurotoxicity. SV2A plays a critical role in vesicle dynamics and neurotransmitter release [74], 75], making it a particularly sensitive indicator of early synaptic dysfunction. Tat has been shown to preferentially impact presynaptic compartments by inducing loss of presynaptic terminals via NMDA receptor-dependent excitotoxicity [76], inhibit VMAT2-mediated dopamine uptake [67], increase oxidative stress and mitochondrial dysfunction [77], 78], and to trigger neuroinflammatory signaling through ROS-mediated NLRP3 inflammasome activation in microglia [79], all of which can impair synaptic integrity. In contrast, PSD-95, as a relatively stable postsynaptic scaffolding protein, may exhibit delayed or region-specific alterations that emerge only at later stages of disease progression [80]. Collectively, these findings suggest that SV2A represents a more immediate and dynamic biomarker of Tat-related synaptic injury in neuroHIV.

The apparent discrepancy between the PET imaging and Western blot findings with respect to sex-specific effects likely reflects both methodological and statistical factors. PET analyses were conducted using a cerebrum VOI, which inherently averages SV2A signal across multiple cortical and subcortical regions. As a result, localized cortical reductions in SV2A, such as those detected by Western blot selectively in female mice, may be diluted when assessed at the whole-cerebrum level, limiting sensitivity to region- and sex-specific effects. Although it is possible to conduct MRI on the same animal before PET and segment the subregions based on registered MR images, the lower resolution (∼1 mm) from PET imaging limits the accurate volume segmentation and quantification. In addition, the small sample size in the PET cohorts reduced power to detect early or sex-dependent differences. In contrast, Western blot analysis provides higher molecular sensitivity and regional resolution, enabling detection of subtle, localized synaptic alterations. Thus, PET imaging captures global, progressive Tat-induced synaptic loss *in vivo*, whereas molecular analyses reveal focal vulnerabilities that may underlie sex-dependent differences in Tat-associated synaptic pathology. Together, these complementary approaches provide a more integrated understanding of Tat-induced synaptic injury across spatial scales. Consistent with this interpretation, cerebrum SUVR values were positively associated with SV2A protein expression in the prefrontal cortex among mice with matched PET and Western blot data, an effect driven by Tat(+) animals. While exploratory, this finding supports the notion that regional cortical vulnerability contributes disproportionately to the global PET signal observed in Tat-expressing mice.

It should be noted that the present study does come with several limitations. First, the sample size per group was limited (n=2–3 per genotype and sex) for PET imaging, which may restrict statistical significance and reduce reproducibility. We used muscle as a reference tissue when calculating SUVR, because no appropriate reference tissue is available within brain region. This is unconventional for CNS tracers. Upon examining the muscle tissue, the time activity curve (TAC) showed low to no uptake in the muscle over the 60 min post injection, except for the small peak during the first couple of minutes, indicating that there was no specific binding in muscle tissue and majority of the signal was from the perfused blood component. Such signal pattern in the muscle stayed the same regardless of DOX treatment, genotype, and sex. These facts suggest that muscle can be used as a reference tissue for the test tracer. We also examined the TAC of the liver. However, there was significant uptake and clearance in the liver. It is unclear whether the uptake was due to nonspecific or specific binding, thus liver tissue was not a good candidate for use as reference tissue. Thus, muscle tissue was chosen as the reference tissue outside of the brain to normalize the brain uptake. Due to the long study duration (three months), it is important to have an internal reference tissue to account for system drifting, errors in system calibration, and errors in injection dose calibration. The major limitation for using muscle tissue is the different blood perfusion levels between muscle and brain. There is an assumption that the perfusion ratio between brain and muscle does not change significantly among genotypes, sex, or DOX treatment. In human imaging, cerebellum is also not used a reference tissue for [^11^C]-UCB-J quantification, instead, white matter region of the centrum semiovale (CS) is chosen as the reference tissue with some limitations due to signal spillover from gray matter [81]. Since white matter in rodent brain is significantly smaller compared to human brain, it makes it impossible to use white matter as the reference tissue in mouse studies. The SUV-to-muscle ratio (SUVR) calculated in this study cannot be directly translated to human imaging because the choice of the reference tissue is different. However, it allows us to compare robustly the effects from genotypes, treatment, and sex within mouse models. Additionally, no kinetic modeling was performed on the dynamic PET data. Although we consider the uptake values at 25 min post injection to provide a valid comparison, given the tracer uptake is relatively stable at this time point, kinetic modeling would offer a more complete characterization of tracer wash-in and wash-out dynamics. Reliable kinetic modeling, however, was not feasible due to limitations in obtaining a blood input function (BIF). For several animals, the heart was positioned outside the imaging field of view, preventing extraction of an image-derived BIF. In other cases, the acquisition did not include the first few minutes, thereby missing the peak of the blood curve. Given these challenges, exacerbated by the difficulty of performing dynamic C-11 imaging while scanning four mice simultaneously, robust kinetic analysis could not be completed. Future work will be needed to optimize the dynamic acquisition procedure to ensure consistent acquisition of an image-derived BIF, which will enable full kinetic modeling. Second, the scope of analyses was restricted to SV2A and PSD-95, which does not fully capture the range of synaptic alterations relevant to HAND. Future studies incorporating additional pre- and postsynaptic proteins (e.g., synaptophysin, synaptotagmin, gephyrin, and AMPA receptor subunits) using complementary methods such as Western blot and immunohistochemistry would provide a more comprehensive view of Tat-induced synaptic alterations. Third, it is important to note that doxycycline (DOX), used to induce Tat expression, has been reported to exert neuroprotective and anti-inflammatory effects [82], 83]. To minimize confounding, both Tat(+) and Tat(−) mice were maintained on DOX-enriched chow throughout the study. It should be pointed out, however, that any influence of DOX would be expected to introduce a conservative bias, potentially attenuating Tat-associated neurobiological changes and leading to an underestimation rather than an exaggeration of the observed effects. Finally, the present study did not include behavioral assessments to directly link Tat-induced synaptic alterations with cognitive or motor outcomes. Ongoing and planned studies are designed to integrate SV2A PET imaging with behavioral measures relevant to HAND-associated impairments, including assessments of learning, memory, and executive function. Such multimodal approaches will be critical for establishing the functional relevance of Tat-associated changes in synaptic density and for strengthening translational links to HAND-related outcomes.

## Conclusions

This study demonstrates that HIV-1 Tat expression in transgenic mice leads to significant reductions in SV2A density, as shown by [^11^C]-UCB-J PET imaging and Western blot analyses, indicating Tat-induced synaptic disruption relevant to HAND. PET imaging captured global, progressive synaptic loss, while Western blot revealed localized, cortical female-specific reductions, highlighting sex- and region-specific vulnerabilities. These findings support the utility of SV2A as a sensitive biomarker of presynaptic dysfunction and underscore its value for detecting early synaptic alterations in neuroHIV. Despite limitations such as small PET sample size, which may have reduced power to detect early or sex-specific effects, the results provide important insights and lay the groundwork for future studies integrating behavioral assessments and therapeutic interventions to preserve synaptic integrity, as well as broader applications of PET imaging in neuroHIV and other neurodegenerative disorders.
